# The complete mitochondrial genome of the subterranean termite, *Reticulitermes speratus kyushuensis* Morimoto, 1968 (Isoptera: Rhinotermitidae)

**DOI:** 10.1080/23802359.2017.1303341

**Published:** 2017-03-28

**Authors:** Wonhoon Lee, Taeman Han, Jong-Ho Lee, Ki-Jeong Hong, Jongsun Park

**Affiliations:** a Department of Plant Medicine and Institute of Agriculture & Life Science, Gyeongsang National University, Jinju-si, Gyeongsangnam-do, Republic of Korea;; b Applied Entomology Division, Department of Agricultural Biology, National Academy of Agricultural Science, Wanju-gun, Jeollabuk-do, Republic of Korea;; c Department of Plant Quarantine, Animal and Plant Quarantine Agency, Gimcheon-si, Gyeongsangbuk-do, Republic of Korea;; d Department of Plant Medicine, College of Life Science and Natural Resources, Sunchon National University, Suncheon-si, Jeollanam-do, Republic of Korea;; e InfoBoss Co. Ltd, Seoul, Republic of Korea;; f InfoBoss Research Center, Gangseo-gu, Seoul, Korea

**Keywords:** Isoptera, mitochondrial genome, *Reticulitermes speratus kyushuensis*, Rhinotermitidae, Korea

## Abstract

We have determined the mitochondrial genome of *Reticulitermes speratus kyushuensis* Morimoto, 1968. The total length of the *R. speratus kyushuensis* is 15,898 bp with 65.3% A + T content. It consists of 13 PCGs, 22 *tRNA*, and 2 *rRNA* genes and an A + T–rich control region. All the protein-coding genes used ATN as start codon. But the stop codons were TAA, TAG, and an incomplete termination codon (T) abutting an adjacent *tRNA* gene. The A + T–rich control region was 1105 bp in length with 67.8% A + T content.

*Reticulitermes speratus kyushuensis* Morimoto, 1968 is an economically important termite species in Korea and has damaged to traditional wooden houses or cultural properties. Although there are some taxonomic studies about this species (Becker 1969; Lee et al. 2015), there is no available information for its mitochondrial genome.

The present study reported the complete mitochondrial genome sequences of *R. speratus kyushuensis*. Samples were collected from Busan, Korea (the specimen is stored in Gyeongsang National University, Korea accession number: GN-160905WH08). Genomic DNA was extracted from soldiers and was sequenced by Illumina HiSeq4000 (Macrogen, Inc., Korea). Raw sequences were filtered by Trimmomatic program and were aligned against the *R. labralis* mitochondrial genome. The mitochondrial genome of *R. speratus kyushuensis* is 15,898 bp in size (accession number KY484910). Its gene content and organization were identical with other *Reticulitermes* species, including 13 protein-coding genes (PCGs), 22 *tRNA* genes, 2 *rRNA* genes and an A + T–rich control region (Cameron & Whiting 2007; Chen et al. 2016; Wang et al. 2016; Zhao et al. 2016). The N chain coded 14 genes, including 8 *tRNA* genes (*tRNAGln*, *tRNACys*, *tRNATyr*, *tRNAPhe*, *tRNAHis*, *tRNAPro*, *tRNALeu*(CUN), and *tRNAVal*), 4 PCGs (*ND5*, *ND4*, *ND4L*, and *ND1*) and 2 *rRNA* genes (*lrRNA* and *srRNA*), and the other 23 genes were coded by the J chain.

The overall sequences in the mitochondrial genome of *R. speratus kyushuensis* were A + T–biased (the G + C content was 34.7%). The PCGs region and A + T–rich region accounted for 64.0% AT and 67.8% AT, respectively. *Reticulitermes speratus kyushensis* had the typical 22 *tRNA* genes throughout the entire mitochondrial genome. All of them, except that *tRNASer*(AGN) lacked the dihydrouridine (DHU) stem, showed the typical clover–leaf secondary structure. Thirteen open reading frames of protein-coding sequences had typical ATN initiation codon. On the other hand, 10 genes had a complete TAA termination codon, whereas 2 genes (*COX2* and *ND5*) showed an incomplete termination codon (T) abutting an adjacent *tRNA* gene, *ND1* had a complete TAG termination codon. This incomplete termination codon is commonly observed in metazoan animals. The *lrRNA* and *srRNA* were located between *tRNALeu*(CUN) and *tRNAVal* and between *tRNAVal* and control region, respectively.

*Reticulitermes speratus kyushuensis* also included intergenic spacers and overlapping regions in common with other termite species. The intergenic spacer sequences were spread on 19 regions ranging in size from 1 to 22 bp, and the overlapping sequences varied from 1 to 6 bp in 5 areas. The A + T–rich control region located between *srRNA* and *tRNAIle* with 1105 bp long and 67.8% A + T content.

Nucleotide sequences of 13 PCGs from 12 closely related species were analyzed to investigate phylogenetic relationships with *R. speratus kyushuensis*. Phylogenetic analysis was performed using maximum likelihood method with 1,000 bootstrap replications with PhyML version 3.0 (Guindon et al. 2010). The phylogenetic tree revealed that *Reticulitermes* was a sister group to *Coptotermes*, and *R. speratus kyushuensis* and *Reticulitermes aculabialis* form a clade (Figure 1). Our study of *R. speratus kyushuensis* will be a useful research for understanding the classification and status in Rhinotermitidae.

**Figure 1.**
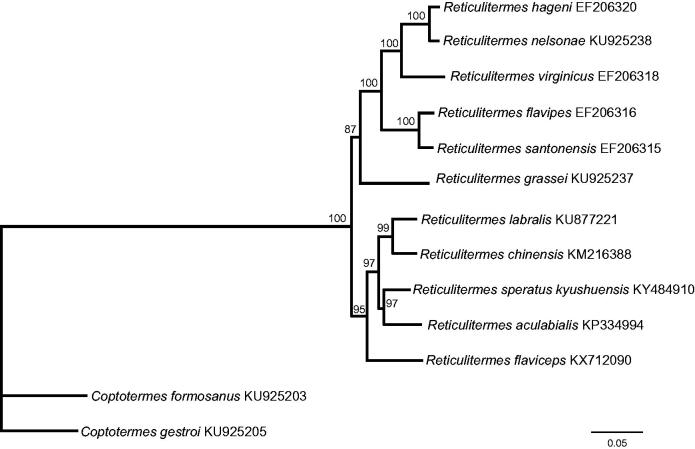
Phylogenetic relationships of two genera *Reticulitermes* and *Coptotermes* based on the nucleotide sequence of 13 PCGs in the mitochondrial genome. The numbers beside the nodes are percentages of 1000 bootstrap values. The *Coptotermes formosanus* and *C. gestroi* were used as an outgroup. Alphanumeric terms indicate the GenBank accession numbers.

## Nucleotide sequence accession number

The complete mitochondrial genome sequence of *R. speratus kyushuensis* has been assigned GenBank accession number KY484910.
